# Method development for simultaneous estimation of Amlodipine Besylate and Perindopril Tertbutyl amine in fixed-dose

**DOI:** 10.1016/j.heliyon.2023.e14209

**Published:** 2023-03-01

**Authors:** Muhammad Farooq Saleem Khan, Lutafullah Tahir, Xu Zhou, Ghulam Bary, Muhammad Sajid, Ahmad Khawar Shahzad, Ilyas Khan, Abdullah Mohamed, Riaz Ahmad

**Affiliations:** a Faculty of International Applied Technology, Yibin University, Yibin 644000, Sichuan China; b Department of Chemistry Minhaj University, Lahore Pakistan; c Faculty of Science, Yibin University, Yibin 644000, Sichuan, China; d Faculty of Materials and Chemical Engineering, Yibin University, Yibin 644000, Sichuan, China; e PUTRA Business School, UPM, Malaysia; f Department of Mathematics, College of Science Al-Zulfi, Majmaah University, Al-Majmaah 11952, Saudi Arabia; g Research Centre, Future University in Egypt, New Cairo 11835, Egypt

**Keywords:** Amlodipine Besylate (ADB), Perindopril Tertbutylamine (PTBA), Fixed-dose, Method development

## Abstract

The fixed-dose combination of Amlodipine Besylate (ADB) with Perindopril Tertbutylamine (PTBA) drug is used to treat patients with mild-to-moderate hypertension. In recent times researchers are interested to find the efficient analytical method development and validation for the simultaneous determination of ADB and PTBA in a fixed-dose, film-coated tablet. Therefore, the current study was performed with a reverse-phase liquid chromatography method developed to simultaneously analyze ADB and PTBA in film-coated tablets as fixed-dose combinations. The linearity of the proposed method was calculated by preparing six different mixtures of both ADB and PTBA in the mobile phase. The concentration of both the analytes was analyzed at 56mg/100 mL to 84mg/100 mL and 32mg/100 mL to 48mg/100 mL, respectively. The ratio of acetonitrile and phosphate buffer was 35:65. The flow rate was adjusted to 1.5 ml per minute to reduce the retention time. The validation study was performed for the parameters specificity, linearity, precision, range, limit of detection, limit of quantification, accuracy/biasness, and robustness. The relative percentage standard deviation for Perindopril Tertbutyl amine was 0.148%, and for Amlodipine is 0.312%. These results show that the advanced analysis method for simultaneous analysis of fixed-dose is precise. The theoretical IR spectra were also calculated by Gaussian 9.2 by employing the B3LYP functional at density functional theory (DFT) level study. All these parameters studied in this work authenticate the effectiveness of the developed validation method and ensure its repeatability/reproducibility accordingly. To the best of our knowledge, this is the first time to develop a new fast, and easy method for simultaneous identification and quantification of ADB and PTBA by high-performance liquid chromatography (HPLC) with a time-efficient and cost-effective approach.

## 1 Introduction

During the last few decades, due to high blood pressure and hypertension, the risk of death due to heart disease has increased. This risk is more prominent in older patients and younger people; this risk has not been established lucidly [1]. The cross-sectional survey data of India exhibited that the average age of the younger patient was 49 years for stage 1 and stage 2 hypertension. So it is necessary to treat younger patients with drugs that proved efficacious in dropping blood pressure to diminish the death risk, caused due to heart diseases [2,3].

Both Amlodipine Besylate (ADB) and Perindopril Tertbutyl amine (PTBA) are used to treat patients suffering from high blood pressure. Perindopril is an inhibitor that inhibits the Angiotensin-Converting Enzyme. It is employed alone or in combination with other drugs to treat patients with high blood pressure [4].

**Fig. 1:**
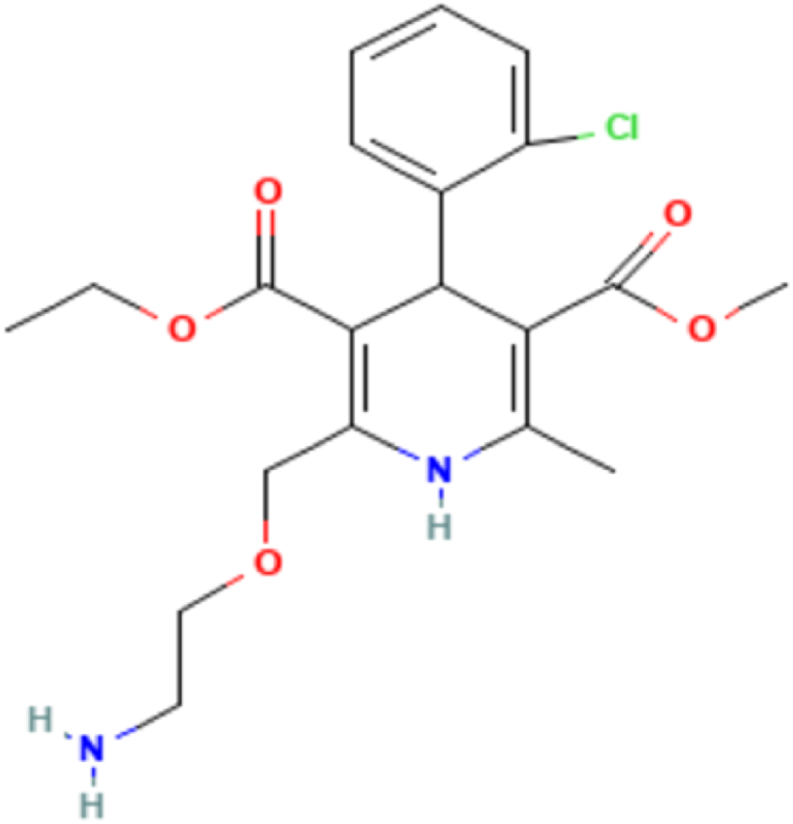
Molecular structure of Amlodipine.

**Fig. 2:**
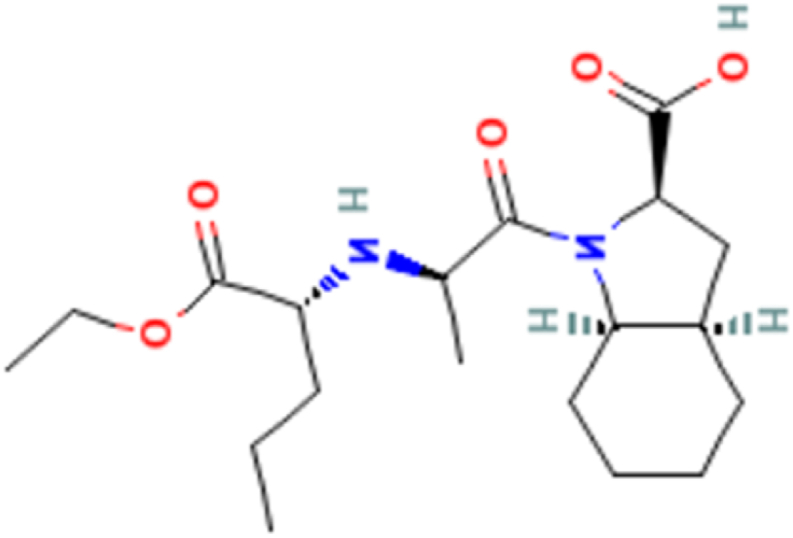
Molecular structure of Perindopril tert-butylamine.

ADB (Fig. 1) belongs to a group of drugs that are called calcium channel blockers. It is also used exercised to treat patients suffering from high blood pressure and a heart disease called angina. It relegates the risk of a heart attack. By using Amlodipine, the blood pressure gets lower, the heart muscles are relaxed, and cardiac arteries are dilated to avoid the blockage of blood vessels; hence, preventing heart attack. Chemically ABD is “3-ethyl 5-methyl 4RS-2-[(2-aminoethoxy) methyl]-4-(2-chlorophenyl)-6-methyl-1,4- dihydropyridine-3,5- dicarboxylate benzene sulphonate" (Figure-1), and PTA is “2- Methyl Propane-2-amine (2S,3As,7As)-1-[(2S)-2- 2[[(1S)-1-(ethoxycarbonyl) butyl]amine] propanoyl]octahydro-1H-indol-2-carboxylate” (Figure-2) [2,5].

Previous data divulged that 51% of deaths are caused by high blood pressure, 45% by heart diseases and coronary heart diseases are due to hypertension. Calcium channel blockers like ADB and angiotensin II receptor blockers like valsartan are used to treat high blood pressure [6]. Combining these two drugs as a single dosage is becoming popular day by day to achieve better patient adherence to treatment and reduce the pill load for patients [7]. During a fixed-dose combination continuation study, the continuation of ramipril treatment was 271 days, and on lisinopril, the continuation of therapy was 211 days out of 360 days [8]. There was a significant difference in adherence of patients to their treatment for hypertension when we changed the combination of drugs to be used for hypertension [9,10]. The adherence of patients with a fixed-dose combination of ramipril and Amlodipine is more favorable than a fixed-dose combination of lisinopril and Amlodipine [5,11].

In recent years, combination therapy has been proven effective in controlling hypertension compared to single-pill drugs. It has also widely improved the patient's compliance with relief. The researchers suggested that the combination drug or polypill be prescribed instead of multiple medications [12]. Some studies suggested liquid chromatography (LC) with mass spectrometry (MS) method by using electrospray ionization mode (EIM) as a good tool for development and validation at a simultaneous determination of, Amlodipine, valsartan (VAL) [12,13].

There are more useful methods reported to monitor drug delivery to targeted places. These methods employed the dose in combining the medicine with biologically suitable magnetic nanoparticles [14]. During the cartilage repair process blood flow into the space in the Synovial joints and can cause severe damage to the joint. The dilution of blood with Synovial fluid can modify the biophysical properties of the blood [15]. To decrease blood viscosity, the technique of laser radiation showed a significant role in the treatment of various viral and auto-immune diseases [16].

The demand for analysis of compounds has increased in low concentrations these days. This has gained importance, particularly in different fields of research and development of new drugs, including synthesis of new medicines, proposed therapy of drugs, differentiation of structure of other compounds, and analysis of these compounds [17,18]. Therefore, it is necessary to prepare a substance with the highest purity, separates the required compounds from impurities, or eliminate unwanted components. So currently, “instrumental analysis” including simple measurements and the preparation of the sample, performing of analysis, and evaluation of the report of samples has also attained significance. This process has become part of “instrumental analysis”. The market claims based on quality issues have increased which makes the products free of complaints. The requirement for the analysis of more samples in a short time is in demand. To meet the market demand, progress has been made in reducing the sample volumes, automation, and thorough screening. The knowledge of techniques used and information obtained from the analysis is necessary to have correct and reliable results [2].

Only four reported methods describe the simultaneous analysis of ADB in pharmaceutical preparations in the literature. LC-MS/MS, HPLC/PDA, HPLC, and HPLC/UV methods. These methods have some limitations in consuming more time and complex analysis. Therefore, more methods are needed to be developed with more efficiency, sensitivity, and swift analytical methods for validation [19,20,21].

It is used as an angiotensin-converting enzyme (ACE) inhibitor, which is long-acting in treating high blood pressure, and heart failure [22]. In the human body, Perindopril tertbutyl amine is metabolized to perindoprilat in the liver, perindoprilat is an active metabolite, and its route of extraction is the urine [23].

The literature survey shows that some analytical methods have been developed for spectrophotometric analysis of PTBA. A few analytical reports are available for the study of Perindopril using different analytical techniques employing Liquid Chromatography (HPLC), Liquid chromatography with a combination of mass spectrometry and gas chromatography both bulk analysis and blood plasma samples [10]. During the review of stability studies of Perindopril, an analytical method has been found which is based on reversed-phase HPLC. This analytical procedure was dependent upon pH. Another analytical approach was used during the dissolution of a fixed-dose combination containing PTBA and indapamide [24]. The previously available analytical methods are more complicated, difficult to prepare reagents, and take a longer time for analysis. These methods generate more waste, and efficiency was also not so good.

Different methods are available for the estimation of ADB. These methods have been developed on various instruments (HPLC, HPTLC). The other techniques include spectrophotometric analysis with mass spectrum [25,26].

To the best of our knowledge, this is the first time to develop a new, fast and easy method for simultaneous identification and quantification of ADB and PTBA by high-performance liquid chromatography (HPLC) with a time-efficient and cost-effective approach. A simple, accurate, precise, and stability-indicating HPLC method was developed to simultaneously estimate ADB and PTBA in a fixed-dose combination of pharmaceutical products and validated by ICH guidelines. The developed methods have a great potential to detect the impurities in the targeted pharmaceutical product. The developed method can be used as a routine analysis for the accurate and precise, assay of ADB and BTBA in the finished dosage form of pharmaceutical products.

## 2 Material and method

### 2.1 Chemicals

The chemicals enlisted in Table 1 were used to develop and validate the analytical method for simultaneous determination of Amlodipine and Perindopril *tert*-butyl amine in fixed-dose combination in tablets.

**Table 1:** List of chemicals.

Sr.#	Name of Chemical	Bach Number	Manufacturer	Expiry Date
1.	Potassium Di-hydrogen Phosphate	AM0973673	Merck/Germany	February 28, 2021
2.	Acetonitrile	1840391 626	Merck/Germany	June 30, 2019
3.	Orthophosphoric acid 85%	E2950	Honeywell Germany	
4.	Methanol			
5.	Amlodipine Besylate working standard	18AD023	Cadila Pharmaceutical, India	Jan 2023
6.	Amlodipine Besylate Raw Material	19AD023	Cadila Pharmaceutical, India	Jan 2024
7.	Perindopril *Tert*-Butyl Amin Raw Material	PEA-18002	Aarti Drugs India	March 2023
8.	Perindopril *Tert*-Butyl Amin working standard	565-317-	Zhejian Huahai china	06-10-2020
9.	Amper 10/4 mg Tablets	BQ-0012	Next Pharmaceutical Products, Pakistan	12–20
10.	Amper 5/4 mg Tablets	BS-0003	Next Pharmaceutical Products, Pakistan	09–20
11.	Coversam 10/4 mg tablets	B(10)20052	Sevier	11–2020
12.	Coversam 5/4 mg Tablets	B(10)1914	Servier Research and Pharmaceuticals, Pakistan	03–2021

### 2.2 Equipment and apparatus

The equipment listed in Table 2 was used during the research work for the development of an analytical method for the analysis of Amlodipine and Perindopril.

**Table 2:** List of equipment.

Sr.#	Equipment Name	Manufacturer and Model	Specifications
1	Precise Weighing Balance	RADWAG/PS510-R1	S/N: 491373 Max: Capacity: 510 gm Readability: 0.001 gm
2	Analytical Balance	RARADWAG Wagi Elektroniczne Model: AS 220.R1, Made in Poland	S/N432966 Max:220 g d = 0,1 mg 12–16V DC/250 mA + 10′C/+40′C
3	pH Meter	Adwa AD1020,	pH/mV/ISE & Temperature Meter
4	FTIR	Agilent Technologies Cary 630FTIR	**Diamond ATR, Power** 110–240 VAC, 60/50 Hz **Spectral range** KBr 6300–350 cm-1 ZnSe 5100–600 cm -1
5	Filtration Assembly	GS Model NO.: AS20	1/6 HP 220–240V 50HZ 3.4 kG N.W., 23L/MIN, 600mmHG Vacuum degree, 25.5*13.5*17 cm(H*W*L), ROHS
6	Sonication Bath	Elma E 30 H Elmasonic	Heater plus Sonicator
7	HPLC	Agilent Technologies 1260 Infinity II	Pressure range of up to 600 bar with a flow rate of up to 5 mL/min allows the use of almost any column – conventional, sub-2μm-particle, or superficially porous columns.
8	Hot plate with a magnetic stirrer	Barnstead/Thermolyne CIMAREC	Hotplate plus Stirrer

Apparatus, including glassware used for method development and validation, is enlisted in S1.

### 2.3 Development of method

Various trials were tried while developing analytical methods for the quantitative determination of Amlodipine and Perindopril Tertbutyl amine. Method development was carried out using the mobile phase of multiple chemicals and various concentrations to optimize the mobile phase proposed for the analysis of Amlodipine and Perindopril tertbutyl amine as active bulk in the tablet dosage form. Different samples were prepared and run on HPLC under other chromatographic conditions. These chromatographic conditions include a change in the flow rate and composition of the mobile phase. However, some parameters like the temperature of the column oven and wavelength were kept constant during the method development process. The design of the mobile phase and other chromatographic systems tried during the study to develop methods for simultaneous analysis of Amlodipine and Perindopril Tertbutyl amine are given in S2.

After many trials, a combination of phosphate buffer pH 2.6, and acetonitrile was selected as the mobile phase for further analysis of Amlodipine and Perindopril Tertbutyl amine and during the execution of the validation study. A different process was performed while developing an analytical method for the simultaneous determination of Amlodipine and Perindopril Tertbutyl amine in pharmaceutical film-coated tablet dosage forms. Preparation of solutions and mobile phase, validation methods, and verification of developed analytical method.

### 2.4 FTIR instrumentation

FTIR analyses were carried out on IR Affinity-1 (00722) FTIR spectrophotometer (Shimadzu, Japan) with IR Solution 1.50 version of the software for data analysis DLATGS detector with a ceramic light source of high luminance Casian, [27]. The experiment was performed with a recording of KBr disc spectra in mid-IR regions 4000cm-1 and 400cm-1, with 45 scans having a resolution of 4cm-1.

### 25 Methods of quantum chemical studies

Quantum chemistry (quantum mechanical calculations) has been done with GaussView 05 software and Gaussian 09 from Gaussian, Inc. USA, to produce the optimized molecular structures [28]. The IR spectra have been calculated by optimizing the frequency of each compound by employing Density Functional Theory (DFT). Then the 6–31 G (d,p) basis set with Becke 3-parameter-Lee-Yang-Parr (B3LYP) functional was also used for simulation [29,30].

## 3 Results and discussions

Amlodipine besylate is a member of the medicines known as a calcium channel blocker. It relaxes the blood vessels, and as a result of relaxing blood vessels, blood can flow easily and hence help lower the blood pressure. It is also used as preventive medicine for some symptoms of chest pain known as angina. Perindopril is an angiotensin-converting enzyme inhibitor used to treat hypertension, heart failure, and other related symptoms. It has two different salts, perindopril tertbutyl amine, and perindopril arginine. Both have the same therapeutic effect; however, there is some difference in the bioequivalence of both salts.

Both Amlodipine and perindopril are used in fixed-dose combinations to treat high blood pressure. Fixed-dose combination can be used to treat high blood pressure and/or stable coronary artery disease in those patients whose conditions are controlled by using these two medicines separately. This combination has reduced the pill load on patients by using one tablet instead of two tablets.

This research work developed a reverse-phase liquid chromatography method to simultaneously analyze Amlodipine and perindopril tertbutyl amine in film-coated tablets as fixed-dose combinations. Validation of this developed method was performed under the guidelines provided by the International Conference of Harmonization (ICH), British Pharmacopeia (B.P.), and the United States Pharmacopeia (USP). The developed method was verified by qualitative and quantitative determination of Amlodipine and perindopril tertbutyl amine in film-coated tablets. The Amper 5/4 mg, Amper 10/8 mg, Coversam 5/4 mg, and Coversam 10/8 mg were analyzed to verify this developed method.

### 3.1 Method development

The analytical method was developed by using different buffers and organic solvents like acetonitrile. Different flow rates were also tried for the simultaneous quantification of both analytes. The tailing factor, additional peaks, and peak symmetry were the major problems. The detail of different chromatographic conditions results is given below in Table 3.

**Table 3:** Results obtained at different chromatographic conditions.

Sr.#	Chromatographic Conditions					Results Obtained from Analysis
	Column	Mobile phase	Flow rate	Injection Volume	Wavelength	
1	Agilent Zorbax Eclipse C18, (Length: 10 cm, dia:4.6 mm particle size 5 μm)	Water 100%	1 ml/min	20 μl	210 mm	Very long retention time and tailing factor
2	Agilent Zorbax Eclipse C18, (Length: 10 cm, dia:4.6 mm particle size 5 μm)	Water: Methanol 50:50% V/V	1 ml/min	20 μl	210 mm	Poor Peak symmetry with long retention time and tailing factor
3	Agilent Zorbax Eclipse C18, (Length: 10 cm, dia:4.6 mm particle size 3.5 μm)	Water: Acetonitrile 50:50% V/V	1.5 ml/min	20 μl	210 mm	Poor peak symmetry and tailing factor
4	Agilent Zorbax Eclipse C18, (Length: 10 cm, dia:4.6 mm particle size 3.5 μm)	Buffer: Acetonitrile 85:15% V/v	1.5 ml/min	20 μl	210 mm	Poor peak Symmetry
5	Agilent Zorbax Eclipse C18, (Length: 10 cm, dia:4.6 mm particle size 3.5 μm)	Buffer: Acetonitrile 70:30% V/v	1.5 ml/min	20 μl	210 mm	Tailing Factor
6	Agilent Zorbax Eclipse C18, (Length: 10 cm, dia:4.6 mm particle size 3.5 μm)	Buffer: Acetonitrile 65:35% V/v	1.5 ml/min	20 μl	210 mm	Fine peak symmetry and tailing factor <2

Water was used in more significant concentrations in the initial trials, and separation was poor. The peaks had low symmetry and a long tailing factor, and the retention time was very long about 30 min. Amlodipine is slightly soluble in water, so separating Amlodipine from the sample was difficult and took a long time with poor peak symmetry.

The combination of organic solvents like methanol with water makes the separation of perindopril easy, but with this combination, the separation of Amlodipine was still not good. Amlodipine is freely soluble in acidic pH, so a buffer at lower pH with organic solvent was tried. Methanol was replaced with acetonitrile, and better symmetry of peak was observed during analysis. In later trials, water was replaced with some buffers at lower pH, and better separation was observed.

The short retention time was achieved by using different combinations of buffer and acetonitrile. The injection volume of 20Ul was kept constant throughout the trials. The flow rate was tried between 1 ml/min to 1.5 ml/min.

The best results were observed by using the combination of phosphate buffer at pH 2.6 and acetonitrile at wavelength 210 nm. The ratio of acetonitrile and phosphate buffer was 35:65. The flow rate of 1.5 ml per minute was adjusted to reduce the retention time. The typical chromatogram is shown in S3.

The retention time for Perindopril tertbutyl amine was about 1.3 min, and for Amlodipine was about 2.3 min. The developed method is time-saving as the retention time for both the analytes is minor than 4 min and also cost-effective as a combination of phosphate buffer and acetonitrile was used as the mobile phase. A comparison with previous published results show that current method is more efficient (Table 4). From the composition of the mobile phase, it is obvious to know that the preparation of the mobile phase is effortless and is in a ratio of 35:65 (Acetonitrile and Phosphate buffer).

**Table 4:** A comparison of retention time of current study with previous studies.

Analyte	Retention time (Min)	Referenece
Perindopril Erbumine	3.172	[31]
Amlodipine Besylate	5.504	[31]
Amlodipine	7.69	[32]
perindopril	7.31	[33]
Perindopril tertbutyl amine	1.3	Current study
Amlodipine	2.3	Current study

The results obtained from replica injections of amlodipine and perindopril tertbutyl amine are presented in Table 5. The concentration of perindopril tertbutyl amine was 0.4 mg/ml, and that of Amlodipine was 0.7 mg/ml.

**Table 5:** Results obtained from Replicate Injections of Standard Solutions.

Perindopril Tertbutyl Amine			Amlodipine		
Concentration	Replicate	Peak Area	Concentration	Replicate	Peak Area
0.4 mg/ml	Replicate-1	5421.371	0.7 mg/ml	Replicate-1	17548.05
	Replicate-2	5422.762		Replicate-2	17432.01
	Replicate-3	5423.347		Replicate-3	17406.862
	Replicate-4	5432.419		Replicate-4	17454.905
	Replicate-5	5440.034		Replicate-5	17435.893
	Replicate-6	5438.538		Replicate-6	17453.315
Mean		5429.745	Mean		17455.173
Standard Deviation		8.022	Standard Deviation		54.470
% Standard Deviation		0.148	% Standard Deviation		0.312%

The table shows that the relative percentage standard deviation for Perindopril Tertbutyl amine is 0.148%, and for Amlodipine is 0.312%. This shows that the advanced analysis method for simultaneous analysis is precise and can be used to analyze tablets containing a fixed-dose combination of Amlodipine and Perindopril Tertbutyl amine in different compositions and strengths.

### 3.2 Analytical method validation

The development of analytical methods and their validations are continuous and interdependent procedures used to analyze newly developed pharmaceutical products and other chemical entities. Validation provides the confidence that an analytical approach will produce reproducible results without bias errors under a given set of pre-defined conditions. The validated method produces reproducible results using the same or different laboratories and persons on other reagents and analytical equipment.

Here, an analytical method for the simultaneous determination of amlodipine and perindopril tertbutyl amine was developed and validated per ICH, BP, and USP guidelines. The validation study was performed for the parameters.

• Specificity
• Linearity
• Range
• Precision
• Limit of detection (LOD)
• Limit of Quantification (LOQ)
• Accuracy/Biasness
• Robustness

#### 3.2.1 Specificity

**Fig. 3:**
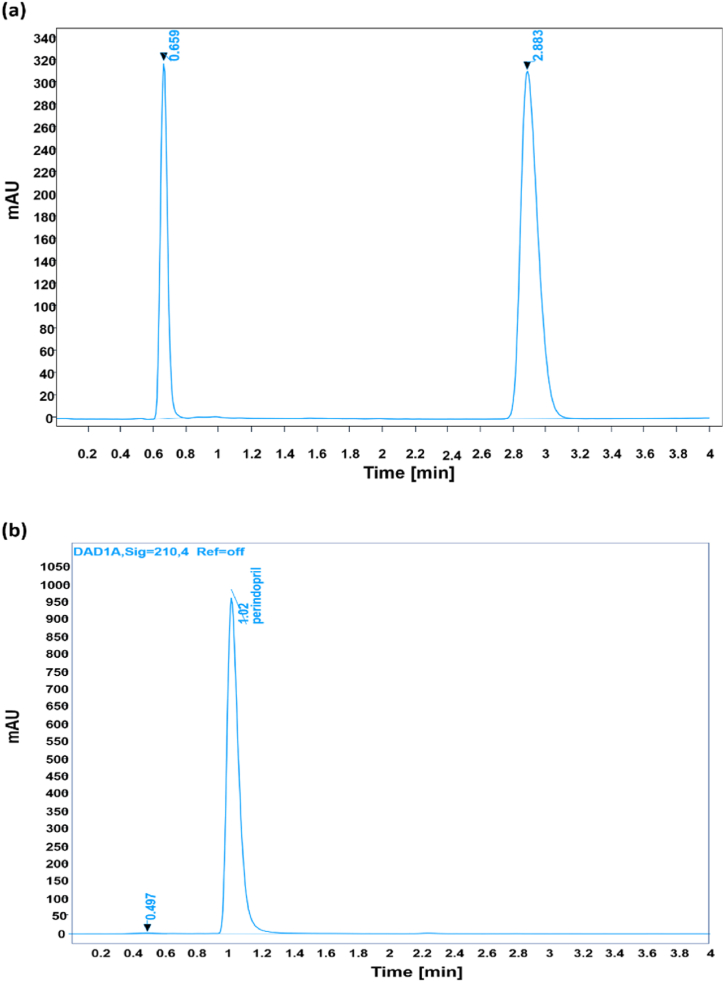
Specificity chromatogram for (a) Amlodipine Besylate and (b)Perindopril tertbutyl amine.

The specificity/selectivity of this advanced method was confirmed by using the sample containing both the analytes and the placebo. The analyte sample gives the specific peak at a particular retention time. This retention time is the qualitative indication and identification of a particular analyte. When a placebo was run under the same chromatographic conditions, no peak was observed. However a sample containing the particular analytes, distinct peaks at a specific retention time were observed for ADB (Fig. 3a) and PTBA (Fig. 3b). The obtained peaks for Standard preparations and sample preparations corresponded to each other, depicting that the developed method was specific for these two particular analytes.

#### 3.2.2 Linearity

**Fig. 4:**
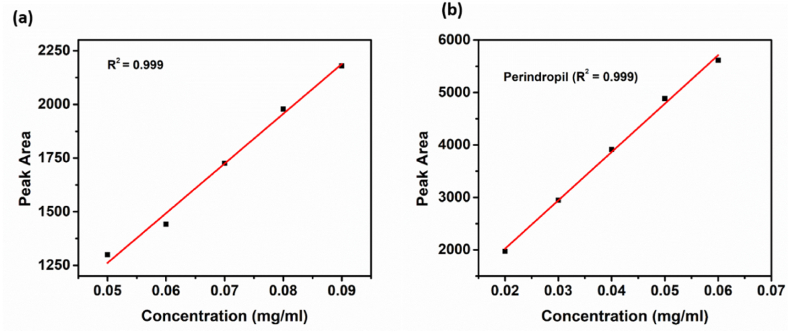
Linearity for (a) Amlodipine Besylate (as a single Analyte) and (b) Perindopril Tertbutyl Amine (as a single Analyte).

Linearity is a relationship between two variables that are directly dependent upon each other. A straight line was obtained when we plot a graph between these two variables. The linearity of an analytical procedure is its ability to show the results directly proportional to the analyte concentration in the given sample. The linearity also limits analytical methods for accurate measurement of the compound under analysis. Linearity can be measured by preparing different concentrations of solutions from standard stock solutions or adding the different weights of analyte into the diluent directly. Therefore, a graph was plotted between the concentration of analyte and peak areas obtained during analysis. The calibration curve was drawn between the analyte concentration and the peak area's mean. The correlation coefficient was calculated, and it was found to be greater than 0.990. The linearity results for ADB (Fig. 4a) and PTBA (Fig. 4b) showed value of R^2^ as 0.999.

During the validation of this method, the linearity for individual analytes was measured by making solutions of different concentrations. The combined linearity is measured by dissolving different weights of both analytes directly into the mobile phase.

##### 3.2.2.1 Linearity for Perindopril Tertbutyl Amine and Amlodipine Besylate (mixture of both analytes)

**Fig. 5:**
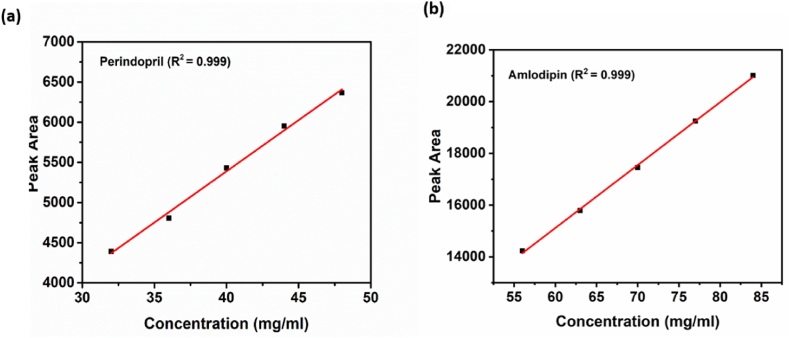
Linearity for (a) Perindopril Tertbutyl Amine and (b) Amlodipine Besylate (Mixture of both analytes).

The linearity of the developed method for Perindopril Tertbutyl Amine (from the mixture) was measured at various concentrations. The results show a correlation coefficient of 0.99 for experimental data of ADB (Fig. 5a) and PTBA (Fig. 5b). Similarly, the linearity of the developed method for Amlodipine Besylate (mixture) was measured at various concentrations. The results show a correlation coefficient of 0.99 for experimental data.

### 3.3 Range

#### 3.3.1 Range for amlodipine Besylate

The developed method has shown that the range of determination of Amlodipine Besylate, as a single analyte, is from 0.05 mg/ml to 0.09 mg/ml, and a linear curve was obtained. The determination range of amlodipine besylate in the mixture with perindopril is from 0.56 mg/ml to 0.84 mg/ml, and a linear curve was obtained within these ranges.

#### 3.3.2 Range for Perindopril tertbutyl amine

The developed method has shown that the range of determination of Perindopril Tertbutyl Amine, as a single analyte, was from 0.02 mg/ml to 0.06 mg/ml, and a linear curve was obtained. The range of determination of Perindopril Tertbutyl Amine in the mixture with amlodipine besylate was from 0.32 mg/ml to 0.48 mg/ml, and a linear curve was obtained within these ranges.

### 3.4 Precision

The precision of an analytical method is the closeness of results obtained from the different sampling of the same homogenous sample. The precision of an analytical method in current study was determined in terms of repeatability, intermediate precision, and reproducibility.

#### 3.4.1 Repeatability

The repeatability of the developed method was calculated by preparing a solution containing a 0.4 mg/ml concentration of perindopril tertbutyl amine and a 0.7 mg/ml concentration of amlodipine besylate. Six replicates of prepared samples were analyzed, results were calculated (S4), repeatability was calculated, and effects were observed within acceptable limits. The % relative standard deviation of the peak areas of six replicas should be less than 2. The % relative standard deviation of the peak areas for Amlodipine besylate and Perindopril Tertbutyl amine are 0.322 and 0.148, respectively, which are well within the given limits (S5).

#### 3.4.2 Intermediate precision

Intermediate precision is the calculation of the closeness of results by using a particular analytical method, under the given set of conditions, but on different days or by various analysts, or by using another instrument. Intermediate precision is measured in terms of the relative standard deviation of the peak areas obtained during the analysis using HPLC. An acceptance limit for intermediate precision is not more than 3% relative standard deviation per ICH guidelines.

Six replicate injections were used to calculate the intermediate precision on different days. On each day, fresh samples were prepared to obtain the results. The solutions contain 0.7 mg/ml concentration of amlodipine besylate and 0.4 mg/ml concentration of perindopril tertbutyl amine. The results are shown in S6 and S7.

#### 3.4.3 Reproducibility

Reproducibility is used for the standardization of any developed analytical method. The variation in the results obtained from different laboratories and locations were calculated to standardize the analytical process. According to the ICH guidelines, reproducibility is required only when the developed analytical method is to be standardized. So, the precision of this developed method was only expressed in terms of repeatability and intermediate precision.

### 3.5 Limit of detection

**Fig. 6:**
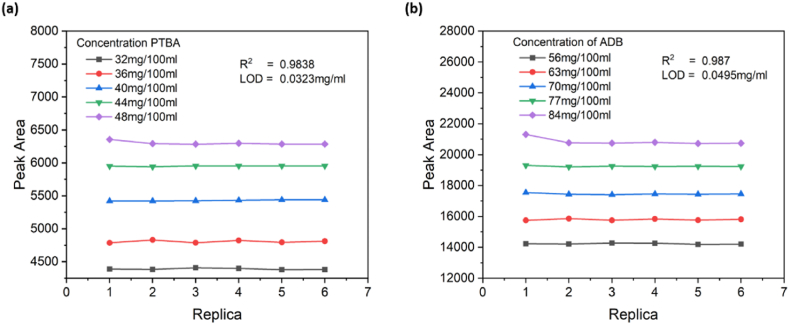
The measurement of the calibration curve of PTBA and ADB at different concentrations (a) PTBA, and (b) ADB.

The limit of detection (LOD) is measured from the calibration curve obtained during the linearity study. Five different concentrations of analytes were used to get the calibration curve. From the curve, the value of the Y-intercept and LOD was calculated. The LOD for PTBA and ADB was observed as 0.0323 mg/ml (Fig. 6a) and 0.0495 mg/ml (Fig. 6b) respectively.

### 3.6 Limit of Quantitation

The limit of Quantitation (LOD) is measured from the calibration curve obtained during the linearity study. Five different concentrations of analytes are used to get the calibration curve. The value of the Y-intercept is calculated from the curve, and LOQ is calculated. The Perindopril Tertbutyl Amine quantification limit was observed as 0.0979 mg/ml, and the correlation coefficient was 0.9838. Whereas the limit of quantification of Amlodipine Besylate was observed as 0.1499 mg/ml, and the correlation coefficient was 0.9876.

### 3.7 Accuracy

The accuracy of an analytical method is measured to calculate the closeness of results obtained, from a developed method, with the accepted reference targets. Different samples are prepared by adding the known different amounts of analytes. Then these samples are analyzed by using the developed analytical method. The percentage recovery of the added known amount is the degree of accuracy of a developed analytical method. Nine measurements over at least three different concentrations were used for accuracy. These prepared concentrations should cover the given specified range. It means three replicate injections of three concentrations. (ICH Guideline 2005) S8. The accuracy of the developed method for both amlodipine besylate and perindopril tertbutyl amine. The mean area of working standard of amlodipine besylate was 17448.036, and for perindopril, tertbutyl amine was 5389.389.

### 3.8 Robustness

The robustness of the developed method was confirmed by making a small but deliberate change in different components of the analytical method, as described in the methodology section. The robustness of the developed method was confirmed analyte ADB (Table 6a) and analyte PTBA (Table 6b).

**Table 6 (a):** Robustness of Amlodipine Besylate.

Replicates	Condition 1		Condition 2		
	Buffer Increase 5%	Buffer Decrease 5%	Area at Standard Temperature 35 °C	Area at -5 °C (30 °C)	Area at +5 °C (40 °C)
Replicate1	17166.419	17073.326	17294.506	17326.728	17337.044
Replicate2	17035.397	17022.539	17297.798	17346.652	17316.671
Replicate3	17014.962	17091.365	17293.112	17315.486	17315.326
Mean	17071.962	17062.410	17295.139	17329.622	17323.014
SD	82.494	35.688	2.406	15.783	12.169
%age RSD	0.48	0.21	0.01	0.09	0.07
**Replicates**	**Condition 3**		**Condition 4**		
	**Flowrate Increase 15%**	**Flowrate Decrease 15%**	**Buffer at** pH **2.62**	**Buffer at** pH **2.51**	**Buffer at** pH **2.70**
Replicate1	14882.024	20284.637	17392.848	17323.788	17350.421
Replicate2	14949.329	20292.762	17394.726	17342.726	17396.573
Replicate3	15028.533	20325.630	17452.222	17337.125	17380.728
Mean	14953.295	20301.010	17413.192	17334.546	17375.907
SD	73.335	21.705	33.811	9.729	23.451
%age RSD	0.49	0.11	0.19	0.06	0.13

**Table 6 (b):** Robustness for perindopril tertbutyl amine.

Replicates	Condition 1		Condition 2		
	Buffer Increase 5%	Buffer Decrease 5%	Area at Standard Temperature 35 °C	Area at -5 °C (30 °C)	Area at +5 °C (40 °C)
Replicate1	5459.751	5334.515	5359.213	5368.406	5370.406
Replicate2	5319.230	5341.326	5360.855	5372.819	5374.390
Replicate3	5218.327	5344.176	5356.705	5369.739	5371.782
Mean	5365.769	5340.005	5358.925	5370.322	5372.192
SD	81.392	4.964	2.090	2.263	2.024
%age RSD	1.52	0.09	0.04	0.04	0.04
**Replicates**	**Condition 3**		**Condition 4**		
	**Flowrate Increase 15%**	**Flowrate Decrease 15%**	**Buffer at pH 2.62**	**Buffer at pH 2.51**	**Buffer at pH 2.70**
Replicate1	4662.012	6304.711	5426.543	5394.580	5398.562
Replicate2	4676.033	6302.388	5425.066	5395.606	5402.662
Replicate3	4678.794	6304.960	5430.654	5391.886	5406.731
Mean	4672.280	6304.019	5427.421	5394.024	5402.652
SD	8.998	1.419	2.896	1.922	4.085
%age RSD	0.19	0.02	0.05	0.04	0.08

### 3.9 Summary of validation parameters

The results of all the parameters used to validate this developed method were within the acceptable range and are shown in S9.

#### 3.9.1 Verification of developed method

The method was developed to simultaneously determine Amlodipine and perindopril tertbutyl amine with a fixed-dose combination in film-coated tablets. This developed method was applied by analyzing different brands and the different strengths of these two analytes. Two additional brands were selected for verification of this developed method. Two strengths of these two brands (four products) were analyzed using this developed method.

#### 3.9.2 Quantitative analysis of Coversam 10/4 mg and Coversam 5/4 mg tablets

The coversam 10/4 mg batch film-coated tablets, Batch Number B(10) 20052, and Coversam 5/4 mg, Batch Number B(10)19114, manufactured by Servier research and pharmaceuticals Pakistan Pvt. Ltd., were analyzed by this developed method. Typical chromatograms of the analysis of these two products are shown in S-10.

#### 3.9.3 Quantitative analysis of AMPER 10/4 mg and AMPER 5/4 mg tablets

AMPER 5/4 mg film-coated tablets, Batch Number BQ0012, and AMPER 10/4 mg film-coated tablets, Batch Number BS0003, manufactured by NEXT Pharmaceutical Products Pvt. Ltd., were analyzed by this developed method. Typical chromatograms of the analysis of these two products are shown in S-11.

### 3.10 System suitability

System suitability tests are performed to verify the performance of analytical equipment, HPLC, as a whole. This verification helps conclude that equipment, operations, electronic system, and samples were analyzed to constitute a system as a whole and work as an integral system. When system suitability parameters are within specified limits, the confidence and reliability of the system enhance.

The following parameters were considered during the verification of this developed method.

• Resolution
• Column efficiency
• Tailing factor

### 3.11 Verification of IR spectra by experimental and theoretical analysis

#### 3.11.1 Amlodipine

**Fig. 7:**
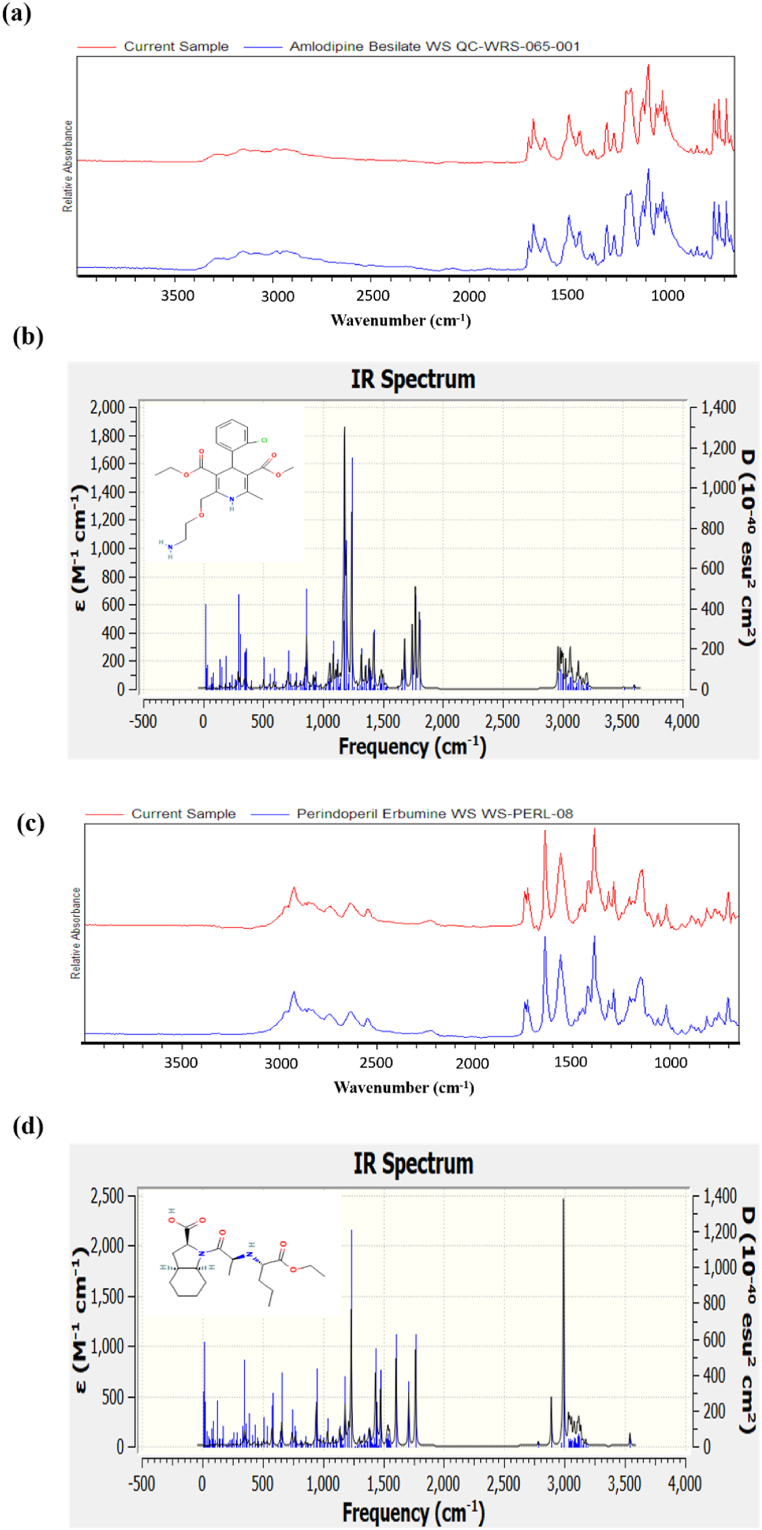
Experimental and theoretical IR spectra (a) experimental spectra of amlodipine (b) theoretical spectra of amlodipine and (c) experimental spectra of perindopril tert-butylamine, and (d) theoretical spectra of perindopril tert-butylamine.

The FTIR spectrum of amlodipine besylate shows absorbance bands in the range of (750, 1715, 1650, 1697, 1735, 1375, 3500-3100, 1100, 3500-3100, 1600-1450, & 1300-1000 cm^−1^). The data shows absorbance band related to the carbonyl group (C=O) as ranging from 1731 to 1687 cm^−1^ for the samples of the Amlodipine with potassium bromide (KBr) and was found to be within 2.0 units of absorbance. The height of peak intensity for the peak range of 1731-1687 cm^−1^ for ADB (Fig. 7a) and PTBA (Fig. 7c) was used to calibrate the curve. It is to be mentioned that the calibration curve for the data results was calculated by equation *y* = c + *bx*, where (.) characterizes peak area and (*x*) characterizes concentration of Amlodipine. The FTIR spectrum specifies that there was negligible interference of excipients used to design a tablet dosage system Casian [18]. In addition, the theoretical data was also calculated by Gaussian 9.2 by employing the B3LYP functional at density functional theory (DFT) level [34]. The theoretical IR spectra are in line with experimental results for ADB (Fig. 7b) and PTBA (Fig. 7b) [35]. The ΔE_gap_ was calculated by low energy unoccupied molecular orbital (LUMO) and high energy molecular orbital (HOMO). The energy gap corresponds to the absorption; therefore, experimental amlodipine data were compared with theoretical calculations in line with experimental data.

#### 3.11.2 Energy calculations of amlodipine

**Fig. 8:**
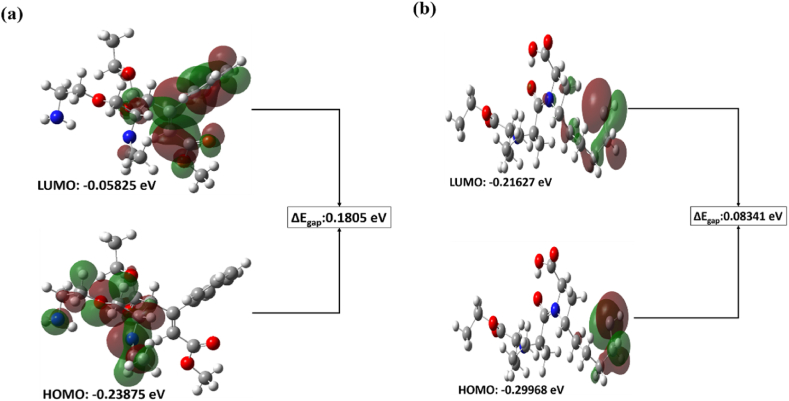
HOMO and LUMO energy gap of (a) ADB and, (b) PTBA.

The experimental, analytical technique Fourier Transform Infrared (FTIR) spectroscopy was used to measure Perindopril Tertbutylamine (drug) spectra. The data were analyzed to find out any interactions between Perindopril and Amlodipine. The theoretical IR spectra were also calculated by Gaussian 9.2 by employing the B3LYP functional at density functional theory (DFT) level study (Fig. 8a). The theoretical results are in line with the experimental data. It can be concluded that there was no significant change in the characteristic peaks of the drug in combination.

The experimental, analytical technique Fourier Transform Infrared (FTIR) spectroscopy was used to measure Perindopril Tertbutylamine (drug) spectra. The data were analyzed to find out any interactions between Perindopril and Amlodipine. The theoretical IR spectra were also calculated by Gaussian 9.2 by employing the B3LYP functional at density functional theory (DFT) level study (Fig. 8b). The theoretical results are in line with the experimental data. It can be concluded that there was no significant change in the characteristic peaks of the drug in combination.

## 4 Conclusion

The developed method has been validated per international conference of harmonization (ICH) guidelines. The validation study was performed for the parameters specificity, linearity, range, precision, limit of detection (LOD), Limit of Quantitation (LOQ), accuracy, and robustness for both analytes. The developed method gives the linear response, and a calibration curve was observed linear in the range of 0.56 mg/ml to 0.84 mg/ml for Amlodipine and in the range of 0.32 mg/ml to 0.48 mg/ml for perindopril tertbutyl amine. The precision of this developed method was calculated in terms of repeatability, and intermediate precision and value were observed precisely, having a relative standard deviation < 2 for both the analytes. LOD and LOQ were 0.0495 mg/ml and 0.1499 mg/ml for Amlodipine besylate whereas 0.0323 mg/ml and 0.0979 mg/ml for perindopril tertbutyl amine. The percentage of recovery was measured by spiking 80%, 100%, and 120% of both the analytes in placebo, where the accuracy was calculated based on the recovery of these analytes. The percentage recovery of Amlodipine was 99.03% to 100.71%, and for perindopril, tertbutyl amine was 97.62% to 102.1%. After validation, this developed method was verified by analyzing two registered products with two different strengths, COVERSAM AM and AMPER. Very accurate results were obtained with a similar peak and retention time pattern without any interference from excipients. These results showed that a new fast and easy method was employed for simultaneous identification and quantification of ADB and PTBA by HPLC with a time-efficient and cost-effective approach. However, the currently proposed method shows some limitations such as the use of sophisticated laboratory instruments (HPLC) and conducting experiments at a standard temperature of 35 °C. So, this developed method can be used to quantitatively determine amlodipine and perindopril tertbutyl amine in finished dosage form film-coated tablets.

## Author contribution statement

Muhammad Farooq Saleem Khan: Conceived and designed the experiments; Analyzed and interpreted the data; Contributed reagents, materials, analysis tools or data; Wrote the paper.

Lutafullah Tahir: Performed the experiments; Contributed reagents, materials, analysis tools or data; Wrote the paper.

Xu Zhou: Conceived and designed the experiments; Analyzed and interpreted the data.

Ghulam Bary: Analyzed and interpreted the data.

Muhammad Sajid, Ahmad Khawar Shahzad, Riaz Ahmad: Analyzed and interpreted the data; Wrote the paper.

Abdullah Mohamed: Conceived and designed the experiments.

Ilyas Khan: Conceived and designed the experiments; Wrote the paper.

## Funding statement

This work was supported by the Natural Science Foundation of Sichuan Province China [2018JY0327].

## Data availability statement

No data was used for the research described in the article.

## Declaration of interest’s statement

The authors declare no conflict of interest.
