# Supplementary material for: Method development for simultaneous estimation of Amlodipine Besylate and Perindopril Tertbutyl amine in fixed-dose
Source: Heliyon. 2023 Mar 1;9(3):e14209. doi: 10.1016/j.heliyon.2023.e14209 (PMC10009730; doi:10.1016/j.heliyon.2023.e14209)
Supplement: Multimedia component 1 [file mmc1.docx]

**Method development for simultaneous estimation of Amlodipine Besylate and perindopril Tertbutyl amine in fixed-dose**

Muhammad Farooq Saleem Khan^1*^, Lutafullah Tahir^2^, Xu Zhou^1^, Ghulam Bary^3^, Muhammad Sajid^4^, Riaz Ahmad^3^

^1^Faculty of International Applied Technology, Yibin University, Yibin 644000, Sichuan China

^2^Department of Chemistry Minhaj University, Lahore Pakistan

^3^Faculty of Science, Yibin University, Yibin 644000, Sichuan, China

^4^Faculty of Materials and Chemical Engineering, Yibin University, Yibin 644000, Sichuan, China

*Corresponding Author:

Email: MFS. Khan [farooqybu@yibinu.edu.cn](mailto:farooqybu@yibinu.edu.cn)

**Supplementary Material**

**S1: Apparatus and glassware**

Table 1: Apparatus and glassware

| **Sr.#** | **Name of Apparatus / Glassware** | **Manufacturer** | **Volume /Specification** |
| --- | --- | --- | --- |
| 1 | Volumetric Flask | Pyrex | Volume: 1000ml |
| 2 | Volumetric Flask | Pyrex | 100ml |
| 3 | Volumetric Flask | Pyrex | 50ml |
| 4 | Volumetric Flask | Pyrex | 25ml |
| 5 | Beaker | Pyrex | 50ml,100ml |
| 6 | Cylinder | Pyrex | 100ml, 500ml, 1000ml |
| 7 | Glass pipette | Pyrex | 5ml, 2ml,1ml |

**S2: Description of mobile phase composition and other chromatographic conditions**

Table 2: Description of mobile phase composition and other chromatographic conditions applied during the development of the method.

| **Sr.#** | **Chromatographic Conditions** | | | | |
| --- | --- | --- | --- | --- | --- |
|  | **Column** | **Mobile phase** | **Flow rate** | **Injection Volume** | **Wavelength** |
| **1** | Agilent Zorbax Eclips C18, (Length: 10cm, dia:4.6mm particle size 3.5 µm) | Water 100% | 1ml/min | 20 µl | 210mm |
| **2** | Agilent Zorbax Eclipse C18, (Length: 10cm, dia:4.6mm particle size 3.5 µm) | Water: Methanol 50:50 % V/V | 1ml/min | 20 µl | 210mm |
| **3** | Agilent Zorbax Eclipse C18, (Length: 10cm, dia:4.6mm particle size 3.5 µm) | Water: Acetonitrile 50:50% V/V | 1.5ml/min | 20 µl | 210mm |
| **4** | Agilent Zorbax Eclipse C18, (Length: 10cm, dia:4.6mm particle size 3.5 µm) | Buffer: Acetonitrile  85:15% V/v | 1.5ml/min | 20 µl | 210mm |
| **5** | Agilent Zorbax Eclipse C18, (Length: 10cm, dia:4.6mm particle size 3.5 µm) | Buffer: Acetonitrile  70:30% V/v | 1.5ml/min | 20 µl | 210mm |
| **6** | Agilent Zorbax Eclipse C18, (Length: 10cm, dia:4.6mm particle size 3.5 µm) | Buffer: Acetonitrile  65:35 % V/v | 1.5ml/min | 20 µl | 210mm |

**S3: Chromatogram of Amlodipine besylate and Perindopril Tertbutyl amine**

1. **Amlodipine besylate**

0

10

20

30

40

50

60

70

80

90

100

110

120

130

140

150

160

170

180

190

mAU

0.659

2.908

DAD1A,Sig=210,4 Ref=off

Figure 1: Chromatogram of amlodipine

1. **Perindopril Tertbutyl amine**

0.2

0.4

0.6

0.8

1

1.2

1.4

1.6

1.8

2

2.2

2.4

2.6

2.8

3

3.2

3.4

3.6

3.8

4

Time [min]

0

50

100

150

200

250

300

350

400

450

500

550

600

650

700

750

800

850

900

950

1000

1050

mAU

0.497

1.02

perindopril

DAD1A,Sig=210,4 Ref=off

Figure 2: Chromatogram of Perindopril Tertbutyl amine

**S4: Repeatability Amlodipine**

Table 3: Calculation of Repeatability for Amlodipine

| **Concentration** | **Replicates** | **Peak Areas** |
| --- | --- | --- |
| 0.7mg/ml | Replica 1 | 17548.05 |
|  | Replica 2 | 17432.01 |
|  | Replica 3 | 17406.862 |
|  | Replica 4 | 17454.905 |
|  | Replica 5 | 17435.893 |
|  | Replica 6 | 17453.315 |
| Mean | | 17455.173 |
| Standard Deviation | | 54.470 |
| % Relative Standard Deviation | | 0.312 |

**S5: Repeatability of Perindopril tertbutyl amine**

Table 4: Calculation of Repeatability for Perindopril tertbutyl amine

| **Concentration** | **Replicates** | **Peak Areas** |
| --- | --- | --- |
| 0.4mg/ml | Replica 1 | 5421.371 |
|  | Replica 2 | 5422.761 |
|  | Replica 3 | 5423.347 |
|  | Replica 4 | 5432.419 |
|  | Replica 5 | 5440.034 |
|  | Replica 6 | 5438.538 |
| Mean | | 5429.745 |
| Standard Deviation | | 8.022 |
| % Relative Standard Deviation | | 0.148 |

**S6: Intermediate precision by Analyst 1/Day1 For Amlodipine Besylate**

Table 5: Finding of intermediate precision by Analyst 1/Day1 For Amlodipine Besylate

| **Replicates** | **Amlodipine Besylate** | | | | | |
| --- | --- | --- | --- | --- | --- | --- |
| **Analyst1/Day1** | **Standard** | **Sample-1** | **Standard** | **Sample-2** | **Standard** | **Sample-1** |
| Replica 1 | 17888.543 | 18661.66 | 18433.383 | 18218.713 | 17892.906 | 18260.401 |
| Replica 2 | 17504.776 | 18488.94 | 17578.247 | 18285.980 | 17775.212 | 18299.849 |
| Replica 3 | 17525.689 | 18516.98 | 17603.091 | 18283.747 | 17887.164 |  |
| Replica 4 | 17625.963 |  | 17577.869 |  | 17951.288 |  |
| Replica 5 | 17531.143 |  | 17558.203 |  | 17977.76 |  |
| Replica 6 | 17557.212 |  | 17568.071 |  | 17967.431 |  |
| Mean | 17605.55 | 18557.86 | 17719.811 | 18262.81 | 17893.627 | 18280.13 |
| SD | 144.850 | 96.118 | 349.897 | 38.208 | 87.343 | 27.894 |
| %RSD | 0.823 | 0.518 | 1.975 | 0.209 | 0.488 | 0.153 |
| mg/Tablet |  | 10.08 |  | 9.99 |  | 10.00 |
| %age Assay |  | 100.80% |  | 99.90% |  | 100% |
|  |  | |  | |  |  |

**S7: Intermediate precision** **by Analyst 1/Day1 For Perindopril Tertbutyl Amine**

Table 6: Finding of intermediate precision by Analyst 1/Day1 For Perindopril Tertbutyl Amine

| **Replicates** | **Perindopril Tertbutyl Amine** | | | | | |
| --- | --- | --- | --- | --- | --- | --- |
| **Analyst 1/Day1** | **Standard** | **Sample-1** | **Standard** | **Sample-2** | **Standard** | **Sample-3** |
| Replica 1 | 5484.42 | 5703.36 | 5562.53 | 5572.688 | 5680.97 | 5676.27 |
| Replica 2 | 5428.07 | 5649.88 | 5441.66 | 5602.882 | 5508.96 | 5690.34 |
| Replica 3 | 5431.04 | 5657.75 | 5446.45 | 5598.235 | 5501.86 |  |
| Replica 4 | 5472.31 |  | 5442.84 |  | 5482.91 |  |
| Replica 5 | 5435.05 |  | 5440.84 |  | 5474.75 |  |
| Replica 6 | 5442.62 |  | 5439.53 |  | 5466.659 |  |
| Mean | 5448.920 | 5670.33 | 5462.308 | 5591.27 | 5519.353 | 5683.31 |
| SD | 23.637 | 28.879 | 49.155 | 16.258 | 80.782 | 9.955 |
| %RSD | 0.434 | 0.509 | 0.900 | 0.291 | 1.464 | 0.175 |
| Mg |  | 8.01 |  | 7.93 |  | 8.02 |
| %age Assay |  | 100.13 |  | 99,13 |  | 100.25 |

**S8:** **Percentage Recovery of Amlodipine Besylate**

Table 7: Calculation of percentage Recovery of Amlodipine Besylate

| **Sr.#** | **Concentration** | **Replicate** | **Peak Area** | **Mean** | **Percentage recovery** |
| --- | --- | --- | --- | --- | --- |
| 1 | 80% Spiked Sample. | Replica 1 | 13349.010 | 13352.320 | 99.03% |
|  |  | Replica 1 | 13362.970 |  |  |
|  |  | Replica 1 | 13344.980 |  |  |
| 2 | 80% Spiked Sample. | Replica 1 | 13342.420 | 13336.870 | 99.15% |
|  |  | Replica 2 | 13331.410 |  |  |
|  |  | Replica 3 | 13336.780 |  |  |
| 3 | 80% Spiked Sample. | Replica 1 | 13460.5 | 13384.730 | 99.27% |
|  |  | Replica 2 | 13355.5 |  |  |
|  |  | Replica 3 | 13338.190 |  |  |
| 4 | 100% Spiked Sample. | Replica 1 | 18163.750 | 18194.700 | 101.20% |
|  |  | Replica 2 | 18205.900 |  |  |
|  |  | Replica 3 | 18214.450 |  |  |
| 5 | 100% Spiked Sample. | Replica 1 | 18283.880 | 18269.133 | 101.6% |
|  |  | Replica 2 | 18268.110 |  |  |
|  |  | Replica 3 | 18255.410 |  |  |
| 6 | 100% Spiked Sample. | Replica 1 | 18348.940 | 18387.377 | 102.1% |
|  |  | Replica 2 | 18394.920 |  |  |
|  |  | Replica 3 | 18418.270 |  |  |
| 7 | 120% Spiked Sample. | Replica 1 | 20125.590 | 20123.907 | 99.5% |
|  |  | Replica 2 | 20117.780 |  |  |
|  |  | Replica 3 | 20128.350 |  |  |
| 8 | 120% Spiked Sample. | Replica 1 | 20118.410 | 20111.660 | 99.4% |
|  |  | Replica 2 | 20078.050 |  |  |
|  |  | Replica 3 | 20138.520 |  |  |
| 9 | 120% Spiked Sample. | Replica 1 | 20069.580 | 20080.417 | 99.3% |
|  |  | Replica 2 | 20084.830 |  |  |
|  |  | Replica 3 | 20086.840 |  |  |

**S9: Summary of the validation Parameters**

Table 8: Summary of the validation Parameters

| **Validation Parameter** | | | **Results** | **Remarks** |
| --- | --- | --- | --- | --- |
| Specificity | | | Chromatograms of the Standard and film coated Tablets containing both analytes were observed comparable. The retention time for both the analytes in film coated tablets correspond to those of obtained in the standard preparation. | So, the developed method for the simultaneous determination of both analytes in fixed dose combination is Specific. |
| Linearity | | Amlodipine Besylate | The correlation coefficient (r^2^) as single analyte is 0.9912 and in the mixture is 0.9992. | The Developed method shows the linear response in the concentration range of 0.05mg/ml to 0.09mg/ml for single analyte and in the range of 0.56mg/ml to 0.84mg/ml in the mixture. |
|  |  | Perindopril Tertbutyl Amine | The correlation coefficient (r^2^) as single analyte is 0.9992 and in the mixture is 0.9954. | The Developed method shows the linear response in the concentration range of 0.02mg/ml to 0.06mg/ml for single analyte and in the range of 0.32mg/ml to 0.48mg/ml in the mixture. |
| Range | | Amlodipine Besylate | The correlation coefficient (r^2^) as single analyte is 0.9912 and in the mixture is 0.9992 and %age standard deviation for replicates of each concentration is <2. | The Developed method shows the reliable results in the concentration range of 0.05mg/ml to 0.09mg/ml for single analyte and in the range of 0.56mg/ml to 0.84mg/ml in the mixture. |
|  |  | Perindopril Tertbutyl Amine | The correlation coefficient (r^2^) as single analyte is 0.9992 and in the mixture is 0.9954 and %age standard deviation for replicates of each concentration is <2. | The Developed method shows reliable results in the concentration range of 0.02mg/ml to 0.06mg/ml for single analyte and in the range of 0.32mg/ml to 0.48mg/ml in the mixture. |
| Precision | | | | |
| 1. Repeatability | | Amlodipine Besylate | The Value of %RSD<2% | The developed method is precise for determination of Amlodipine Besylate in term of repeatability. |
|  |  | Perindopril Tertbutyl Amine | The Value of %RSD<2% | The developed method is precise for determination of Perindopril Tertbutyl Amine in term of repeatability. |
| 1. Intermediate Precision | | Amlodipine Besylate | The Value of %RSD<2% | The developed method is precise for determination of Amlodipine Besylate in term of intermediate precision. |
|  |  | Perindopril Tertbutyl Amine | The Value of %RSD<2% | The developed method is precise for determination of Perindopril Tertbutyl Amine in term of intermediate precision. |
| Limit of detection | Amlodipine Besylate | | 0.0495mg/ml | The Developed method can detect 0.0495mg/ml concentration of Amlodipine besylate in the given solution. |
|  | Perindopril Tertbutyl Amine | | 0.0323mg/ml | The Developed method can detect 0.0323mg/ml concentration of Perindopril Tertbutyl Amine in the given solution. |
| Limit of Quantitation | Amlodipine Besylate | | 0.1499mg/ml | The Developed method can measure 0.1499mg/ml concentration of Amlodipine besylate, quantitatively in the given solution. |
|  | Perindopril Tertbutyl Amine | | 0.0979mg/ml | The Developed method can measure 0.0979mg/ml concentration of perindopril Tertbutyl Amine, quantitatively in the given solution. |
| Accuracy | Amlodipine Besylate | | Percentage recovery by adding 80%, 100% and 120% of analyte in the placebo was in range of 99.03% to 100.71% | The developed method for determination of perindopril tertbutyl amine in the mixture is accurate |
|  | Perindopril Tertbutyl Amine | | Percentage recovery by adding 80%, 100% and 120% of analyte in the placebo was in range of 97.62% to 102.1% | The developed method for determination of Amlodipine Besylate in the mixture is accurate |
| Robustness | | | The %age RSD of Peak areas for both the analytes, after making small deliberate changes is <2. | The developed method is Robust over small deliberate changes in the given set of conditions of defined parameters. |

**S10:** **Analysis of Coversam 10/4mg Film-Coated Tablets**

Table 9 (a): Results of Analysis of Coversam 10/4mg Film-Coated Tablets

| **Product** | **Replicates** | **Amlodipine Sample** | **Amlodipine Standard** | **Perindopril Sample** | **Perindopril standard** |
| --- | --- | --- | --- | --- | --- |
|  | Replica 1 | 17605.140 | 17303.5 | 2516.040 | 2659.940 |
|  | Replica 2 | 17612.380 | 17299.840 | 2531.150 | 2660.340 |
| Mean | | 17608.760 | 17301.67 | 2523.595 | 2660.14 |
| Standard Deviation | | 5.119 | 2.588 | 10.684 | 0.282 |
| % Relative Standard Deviation | | 0.029% | 0.015% | 0.423% | 0.011% |
| Results | | | | | |
| Quantity of Amlodipine per tablet | | **10.17mg** | |  |  |
| Percentage Assay for Amlodipine | | **101.74%** | |  |  |
| Quantity of Perindopril Tertbutyl amine per tablet | | **3.88mg** | |  |  |
| Percentage Assay for Perindopril Tertbutyl amine | | **97.00%** | |  |  |

Table 9(b): Results of Analysis of Coversam 5/4mg Film-Coated Tablets

| **Product** | **Replicates** | **Amlodipine Sample** | **Amlodipine Standard** | **Perindopril Sample** | **Perindopril standard** |
| --- | --- | --- | --- | --- | --- |
|  | Replica 1 | 17166.780 | 17057.100 | 5151.480 | 5272.180 |
|  | Replica 2 | 17175.400 | 17070.040 | 5169.850 | 5205.980 |
| Mean | | 17171.09 | 17063.57 | 5160.665 | 5239.08 |
| Standard Deviation | | 6.095 | 9.149 | 12.990 | 46.810 |
| % Relative Standard Deviation | | 0.035% | 0.053% | 0.252% | 0.893% |
| Results | | | | | |
| Quantity of Amlodipine per tablet | | **5.01mg** | |  |  |
| Percentage Assay for Amlodipine | | **100.31%** | |  |  |
| Quantity of Perindopril Tertbutyl amine per tablet | | **3.93mg** | |  |  |
| Percentage Assay for Perindopril Tertbutyl amine | | **98.24%** | |  |  |

**S11: Analysis of AMPER 10/4mg Film-Coated Tablets**

Table 10 (a): Results of Analysis of AMPER 10/4mg Film-Coated Tablets

| **Product** | **Replicates** | **Amlodipine Sample** | **Amlodipine Standard** | | **Perindopril Sample** | **Perindopril standard** |
| --- | --- | --- | --- | --- | --- | --- |
|  | Replica 1 | 17605.979 | 17307.160 | | 2512.2 | 2659.940 |
|  | Replica 2 | 17604.048 | 17299.840 | | 2527.796 | 2660.340 |
| Mean | | 17605.01 | 17303.56 | | 2519.99 | 2660.14 |
| Standard Deviation | | 1.365 | 5.176 | | 11.028 | 0.2828 |
| % Relative Standard Deviation | | 0.008% | 0.029% | | 0.437% | 0.010% |
| Results | | | | | | |
| Quantity of Amlodipine per tablet | | **10.17mg** | |  |  |  |
| Percentage Assay for Amlodipine | | **101.7%** | |  |  |  |
| Quantity of Perindopril Tertbutyl amine per tablet | | **3.87mg** | |  |  |  |
| Percentage Assay for Perindopril Tertbutyl amine | | **96.83%** | |  |  |  |

Table 10 (b): Results of Analysis of AMPER 5/4mg Film-Coated Tablets

| **Product** | **Replicates** | **Amlodipine Sample** | **Amlodipine Standard** | **Perindopril Sample** | **Perindopril standard** |
| --- | --- | --- | --- | --- | --- |
|  | Replica 1 | 17112.664 | 17057.100 | 5083.169 | 5272.180 |
|  | Replica 2 | 17099.649 | 17070.040 | 5043.564 | 5205.980 |
| Mean | | 17106.16 | 17063.57 | 5063.36 | 5239.08 |
| Standard Deviation | | 9.20 | 9.149 | 28.004 | 46.8104 |
| % Relative Standard Deviation | | 0.053% | 0.054% | 0.553% | 0.893% |
| Results | | | | | |
| Quantity of Amlodipine per tablet | | **4.99mg** | |  |  |
| Percentage Assay for Amlodipine | | **99.93%** | |  |  |
| Quantity of Perindopril Tertbutyl amine per tablet | | **3.86mg** | |  |  |
| Percentage Assay for Perindopril Tertbutyl amine | | **96.39%** | |  |  |

**S12: percentage Recovery**

Table 11: Calculation of percentage Recovery of Perindopril Tertbutyl Amine

| **Sr.#** | **Concentration** | **Replicate** | **Peak Area** | **Mean** | **Percentage recovery** |
| --- | --- | --- | --- | --- | --- |
| 1 | 80% Spiked Sample. | Replica 1 | 4097.893 | 4100.115 | 97.92% |
|  |  | Replica 1 | 4104.880 |  |  |
|  |  | Replica 1 | 4097.573 |  |  |
| 2 | 80% Spiked Sample. | Replica 1 | 4090.385 | 4087.827 | 97.62% |
|  |  | Replica 2 | 4086.118 |  |  |
|  |  | Replica 3 | 4086.977 |  |  |
| 3 | 80% Spiked Sample. | Replica 1 | 4126.402 | 4115.378 | 98.26% |
|  |  | Replica 2 | 4111.035 |  |  |
|  |  | Replica 3 | 4108.696 |  |  |
| 4 | 100% Spiked Sample. | Replica 1 | 5551.061 | 5559.363 | 99.57% |
|  |  | Replica 2 | 5564.155 |  |  |
|  |  | Replica 3 | 5562.872 |  |  |
| 5 | 100% Spiked Sample. | Replica 1 | 5590.738 | 5591.144 | 100.14% |
|  |  | Replica 2 | 5598.171 |  |  |
|  |  | Replica 3 | 5584.522 |  |  |
| 6 | 100% Spiked Sample. | Replica 1 | 5613.076 | 5630.608 | 100.71% |
|  |  | Replica 2 | 5639.778 |  |  |
|  |  | Replica 3 | 5638.972 |  |  |
| 7 | 120% Spiked Sample. | Replica 1 | 6183.447 | 6185.571 | 99.04% |
|  |  | Replica 2 | 6190.928 |  |  |
|  |  | Replica 3 | 6182.338 |  |  |
| 8 | 120% Spiked Sample. | Replica 1 | 6184.665 | 6182.649 | 98.43% |
|  |  | Replica 2 | 6171.930 |  |  |
|  |  | Replica 3 | 6191.352 |  |  |
| 9 | 120% Spiked Sample. | Replica 1 | 6166.918 | 6169.901 | 98.23% |
|  |  | Replica 2 | 6171.128 |  |  |
|  |  | Replica 3 | 6171.656 |  |  |
